# Etiology and Outcome of Patients with HIV Infection and Respiratory Failure Admitted to the Intensive Care Unit

**DOI:** 10.1155/2013/732421

**Published:** 2013-08-28

**Authors:** Jose Orsini, Noeen Ahmad, Ashvin Butala, Rosemarie Flores, Truc Tran, Alfonso Llosa, Edward Fishkin

**Affiliations:** ^1^Department of Medicine, New York University School of Medicine at Woodhull Medical and Mental Health Center, 760 Broadway, Brooklyn, New York, NY 11206, USA; ^2^Division of Critical Care Medicine, Department of Medicine, New York University School of Medicine at Woodhull Medical and Mental Health Center, 760 Broadway, Brooklyn, New York, NY 11206, USA

## Abstract

*Background*. Although access to HAART has prolonged survival and improved quality of life, HIV-infected patients with severe immunosuppression or comorbidities may develop complications that require critical care support. Our objective is to evaluate the etiology of respiratory failure in patients with HIV infection admitted to the ICU, its relationship with the T-lymphocytes cell count as well as the use of HAART, and its impact on outcome. *Methods*. A single-center, prospective, and observational study among all patients with HIV-infection and respiratory failure admitted to the ICU from December 1, 2011, to February 28, 2013, was conducted. *Results*. A total of 42 patients were admitted during the study period. Their median CD_4_ cell count was 123 cells/**μ**L (mean 205.7, range 2.0–694.0), with a median HIV viral load of 203.5 copies/mL (mean 58,676, range <20–367,649). At the time of admission, 23 patients (54.8%) were receiving HAART. Use of antiretroviral therapy at ICU admission was not associated with survival, but it was associated with higher CD_4_ cell counts and lower HIV viral loads. Twenty-five patients (59.5%) had respiratory failure secondary to non-HIV-related diseases. Mechanical ventilation was required in 36 patients (85.1%). Thirteen patients (31.0%) died. *Conclusions*. Noninfectious etiologies of respiratory failure account for majority of HIV-infected patients admitted to ICU. Increased mortality was observed among patients with sepsis as etiology of respiratory failure (HIV related and non-AIDS related), in those receiving mechanical ventilation, and in patients with decreased CD_4_ cell count. Survival was not associated with the use of HAART. Complementary studies are warranted to address the impact of HAART on outcomes of HIV-infected patients with respiratory failure admitted to ICU.

## 1. Introduction

 The rate of admission to the ICU and intensive care mortality in patients with HIV infection have shifted repeatedly during the AIDS epidemic, influenced by attitudes of patients and providers toward utility of care. Respiratory failure remains the most common diagnosis for ICU admission in patients with HIV infection, and, despite advances in medical therapy and intensive care medicine, the mortality of these patients remains substantial [1, 2]. However, the incidence of AIDS-associated illnesses has significantly decreased, and the overall life expectancy of HIV-infected patients markedly increased after the introduction of HAART [3, 4]. Nevertheless, infectious and noninfectious complications that may require critical care support continue to occur in this patient population. In the past decade, the number of patients with AIDS-defining conditions admitted to the ICU decreased dramatically. This decreasing pattern has been offset by a parallel increase in the number of patients with HIV infection admitted for medical problems nonrelated to HIV infection [5]. Several studies have suggested that the introduction of HAART not only improved the survival of HIV-infected patients admitted to ICU but also changed the etiology of admissions of these patients to the critical care units [5–7]. Unfortunately, only 25%–50% of HIV-infected patients receive HAART at the time of admission to the ICU [5, 6]. Retrospective cohort studies have shown that prognostic factors of mortality in HIV-infected patients admitted to ICU are diverse, including severity of acute illness, poor functional status, and respiratory failure requiring mechanical ventilation [8, 9]. 

In this study, the authors evaluated the etiology of respiratory failure among HIV-infected patients requiring ICU admission, its relationship with their CD_4_ lymphocyte count as well as the use of HAART, and its impact on outcome.

## 2. Methods

### 2.1. Study Design and Patient Population

The study has a prospective, observational design. It was conducted in a general, community innercity hospital located in Brooklyn, New York (Woodhull Medical and Mental Health Center), which serves a multiethnic HIV population. The hospital has a total of 346 beds distributed among general internal medicine, general surgery, psychiatry, obstetrics/gynecology, and pediatrics wards. The ICU at our institution works under a “closed system” with the supervision of 5 board-certified intensivists and is composed of 12 beds, with an annual admission average of 700 patients. All patients, 18 years or older, with known HIV infection and respiratory failure admitted to the ICU, either from the emergency department or transfered from the medical or surgical wards, were enrolled in the study. We excluded patients with HIV infection admitted to the ICU for reasons other than respiratory failure. For the purpose of this study, only data from the first admission was analyzed in patients readmitted to ICU within 30 days. Variables such as age, gender, comorbid conditions, Acute Physiology and Chronic Health Evaluation- (APACHE-) II score at the time of ICU admission, T-lymphocytes count (CD_4_), HIV viral load by PCR (test was performed using the COBAS AmpliPrep/COBAS Taqman, HIV-1 test kit version 2.0, Roche Molecular systems, Inc.), use of HAART at the time of admission, needs for mechanical ventilation or noninvasive ventilation and/or vasopressors, and outcome at hospital and ICU discharge were measured. Computerized medical records were reviewed and clinical information was abstracted for each patient. After waiving the need for informed consent, the Institutional Review Board approved the study.

### 2.2. Results

A total of 42 patients with HIV infection and respiratory failure were admitted to ICU during the 15-month study period. On admission to ICU, 23 patients (54.8%) were receiving HAART. Nineteen patients (45.2%) were not on antiretroviral therapy, either because of poor adherence or lack of access to medical care. None of the patients were started on HAART in the ICU. Their median CD_4_ cell count was 123.0 cells/*μ*L (mean 205.7, range 2.0–694.0), with a median HIV viral load of 203.5 copies/mL (mean 58,676, range <20–367,649). Among patients receiving HAART, the median CD_4_ cell count was 187.9 cells/*μ*L (mean 218.5, range 2.0–694.0), with a correspondent median HIV viral load of 51 copies/mL (mean 27,356, and range <20–235,769). Patients not receiving HAART had lower CD_4_ cell count (median 51.0 cells/*μ*L, mean 190.2, and range 3.0–689.6) and higher viral load (median 55,851 copies/mL, mean 96,594, range <20–367,649). The median age was 52 years (range 20–80). Twenty-nine patients (69.0%) were males. On admission to ICU, the median APACHE-II score was 15 (mean 15.9, range 5–27). Patients with noninfectious etiology of respiratory failure had higher APACHE-II score (median 17, range 8–27 versus median 13, range 5–23). Infectious etiology of respiratory failure was identified in 17 patients (40.5%), while a noninfectious reason was accountable in 25 patients (59.5%). Both patients on HAART and those not receiving antiretroviral therapy were most likely to have a noninfectious etiology for the respiratory failure (14, 60.9% and 11, 57.9%, resp.). Etiologies of respiratory failure in these patients are summarized as follows. 

Infectious etiology of respiratory failure in patients with HIV infection is as follows:

1. bacterial pneumonia  (6)
2. *Pneumocystis jirovecii* pneumonia  (4)
3. intra-abdominal sepsis  (3): abscess  (1), biliary tract sepsis  (1), vancomycin-resistant *Enterococcus* (VRE) bacteremia  (1)
4. lung abscess  (1)
5. CNS infection  (2): bacterial meningitis  (1), *Cryptococcus neoformans* meningitis  (1)
6. nasopharyngeal abscess  (1)

Noninfectious etiology of respiratory failure in patients with HIV infection is as follows:

1. decompensated congestive heart failure (CHF) (8),
2. drug overdose  (4),
3. cardiac arrest  (4),
4. electrolyte imbalance  (2),
5. hemorrhagic shock  (2),
6. chronic obstructive pulmonary disease (COPD) exacerbation  (1),
7. status epilepticus  (1),
8. upper airways obstruction (angioedema, 2),
9. thrombotic thrombocytopenic purpura (TTP) (1).

Thirty-six patients (85.1%) required mechanical ventilation, while 2 patients (4.8%) needed noninvasive ventilation. Four patients (9.5%) achieved acceptable oxygen saturation by receiving oxygen via nasal cannula. The total number of ventilator days required was 297 (median 5.5, mean 8.3, and range 1–38). The group of patients with infectious etiology of respiratory failure required mechanical ventilation for longer period (median 7, range 1–38 days versus median 4, range 1–17 days). Both patients on HAART and those not on antiretroviral therapy had comparable length of stay in ICU (median 5, mean 7.8, and range 1–31 days versus median 4, mean 6.8, and range 1–40 days, resp.). Six patients (14.3%) underwent tracheostomy procedures because of prolonged mechanical ventilation dependence. A total of 18 patients (42.9%) required vasoactive therapy, either in the form of vasopressors or inotropes. Thirteen patients (31.0%) died in the hospital. Ten of those patients (76.9%) died in ICU. From those, 4 patients (30.8%) were terminally extubated. Eight patients (61.5%) were receiving HAART at the time of death. There was no significant difference in the APACHE-II scores of survivors and nonsurvivors (median 15, range 8–27 versus median 15, range 5–26, resp.). The median CD_4_ cell count of the patients who did not survived was 27.0 cells/*μ*L (mean 110.3, range 3.7–622.0), and their median viral load was 476 copies/mL (mean 79,015, range <20–367,649). Majority were males (11, 84.6%), and their median age was 50 years (range 20–69). Mortality rate was higher in patients with infectious etiology of respiratory failure (8, 61.5%). The total number of ICU days utilized by all patients was 299 (median 4.5, mean 6.8, and range 1–40).

## 3. Discussion

This study examined the characteristics of HIV-infected patients with respiratory failure admitted to ICU. It provides important insights into the critically ill patient with HIV infection, through identification of variables influencing the outcome of these patients. To our knowledge, this is the first study to analyze the etiology of respiratory failure in HIV-infected patients requiring ICU admission and their outcome in the New York City public hospitals system (New York City Health and Hospitals Corporation).

Since the onset of the HIV epidemic, there has been a significant shift away from AIDS-defining diagnoses as indications for ICU admission. In this study, non-HIV-related illnesses accounted for majority of admissions, which goes in accordance to results from other studies [6, 9, 10]. The overall hospital survival rate in this report is comparable to data previously published [10–12]. Although the reported overall hospital mortality rate in this study was lower than in other series [6, 13, 14], our ICU survival rate was remarkably lower when compared with similar studies [5, 9, 11, 13–15]. Majority of our patients were receiving antiretroviral therapy at the time of death. On the other hand, none of patients that were not receiving HAART were initiated on antiretroviral therapy in ICU. While the long-term benefits of HAART on survival among HIV-infected outpatients are indisputable, there may be no short-term benefits in ICU patients with critical illnesses. Data on the impact of HAART on the incidence of critical illness among HIV-infected patients are limited. HAART leads to decreased opportunistic infections that collectively could contribute to lower risk of ICU admission. Because HIV-infected patients are living longer, however, they are at increasing risk of developing comorbid illnesses not previously thought to be HIV related, and these non-AIDS-related conditions account proportionally for the majority of deaths [15, 16]. Our results failed to demonstrate the potential protective and beneficial effect of HAART in both ICU admission and mortality, contradicting results from previous reports [5, 17–20]. It is well known that the use of HAART is associated with a dramatic reduction in HIV viral load and with a concomitant increase in CD_4_ cell counts. Management of antiretroviral therapy is a challenge in ICU, and introducing HAART in a critically ill patient is a difficult decision. There are no pharmacologic or pharmacokinetic data concerning antiretroviral therapy in hemodynamic unstable patients. Concerns about drug-to-drug interactions and toxicities, medication absorption, and variability of drug levels are common challenges among intensivists. Since the authors of this study did not collect information on adherence to HAART prior to ICU admission and our observational study did not measure HAART resistance, the impact of nonadherence or drug resistance as explanations cannot be excluded. Therefore, our study probably does not reflect the effect that HAART has on the survival of patients with HIV infection admitted to the ICU. In addition, this study does not address the real value of intensive care for critically ill patients with HIV infection, because it is possible that a significant selection bias existed during the triaging process. Although HAART use may not be associated with short-term ICU survival, long-term survival may be improved in the group of ICU survivors who received antiretroviral therapy [5, 20–22]. In one study, Morquin et al. showed that introducing HAART on admission to ICU seems to be protective in both short- and long-term outcomes [20]. Nevertheless, the overall mortality rate in this study falls into the range observed in other series [2, 6, 11, 23]. 

As previously described [9, 11, 20, 24–26], the authors of this study identified independent predictors of hospital mortality: low CD_4_ cell count, infectious etiology of respiratory failure or sepsis, and needs for mechanical ventilation and/or vasopressors. Although comparable to results from other similar studies [12, 14, 27], the median CD_4_ cell count in our patients was higher than those reported in other series [9, 10, 13, 28]. A large proportion of our HIV-infected population is known for poor adherence to antiretrovirals and ongoing substance abuse. The substandard treatment responses to HAART in our patients may be due to the presence of transmitted drug resistance of the HIV strains, poor adherence to HAART that may result in emergence of antiretroviral drug resistance mutations, and suboptimal or nonimmunological response. The median APACHE-II score in our patients was lower than reported in previous studies [10, 14, 18, 21], and we found no significant difference in the APACHE-II score of survivors and nonsurvivors. This result contradicted data from other studies, where elevated APACHE-II scores were prognostic of adverse outcomes in these patients [5, 9, 10, 12]. Interesting is the high number of patients who received mechanical ventilation in this study, outweighing results from other reports [9–11, 13, 14, 20, 23, 28]. However, our patients' length of stay in ICU is comparable to figures shown previously [11, 14, 18, 27]. We hypothesized that our lengthened ICU stay was the result of some patients who developed complications such as acute respiratory distress syndrome and hemodynamic instability during the course of their illnesses and, therefore, weaning from mechanical ventilation and/or tracheostomy procedures were delayed. It may also explain the large number of tracheostomy procedures performed in our patients, when compared to data published by Coquet et al. [26]. In addition, and similar to one study [20], near half of our patients received vasopressors during their ICU stay, which contributed as well to adverse outcomes. These results may be explained by factors related to our patient population and their availability to access medical care. Probably, our patients did not seek medical care at the beginning of their illnesses, which likely is related to suboptimal outcomes.

The main strength of this study is the prospective nature of its design, with evaluation of all consecutive admissions to ICU of patients with HIV infection and respiratory failure. There are some limitations in this study. First, the observational nature limits firm conclusions regarding HAART use and survival. Second, the case number is relatively small and it was conducted in a single center. Therefore, the results may not be generalizable and applicable to other institutions and HIV populations since clinical practice and demographics may differ across institutions. Third, this study does not address outcomes after hospital discharge. Therefore, the predictors of long-term survival and any impact of antiretrovirals on long-term survival remain unknown.

## 4. Conclusions

The ICU utilization for HIV-infected patients in our institution has not been changed by the advent of HAART, and the impact of antiretroviral therapy on the indications and outcome of ICU admission remains controversial. Our data suggested that the short-term survival of HIV-infected patients admitted to the ICU is not dependent on the use of HAART and that outcomes are mainly related to the degree of immunosuppression. Larger prospective studies are needed to examine the impact of HAART and other parameters on the outcomes of patients with HIV infection and respiratory failure admitted to the ICU.

## References

[1] Rosen MJ, Narasimhan M (2006). Critical care of immunocompromised patients: human immunodeficiency virus. *Critical Care Medicine*.

[2] Akgün KM, Pisani M, Crothers K (2011). The changing epidemiology of HIV-infected patients in the intensive care unit. *Journal of Intensive Care Medicine*.

[3] Li TS, Tubiana R, Katlama C, Calvez V, Mohand HA, Autran B (1998). Long-lasting recovery in CD_4_ T-cell function and viral-load reduction after highly active antiretroviral therapy in advanced HIV-1 disease. *The Lancet*.

[4] Palella FJ, Delaney KM, Moorman AC (1998). Declining morbidity and mortality among patients with advanced human immunodeficiency virus infection. *The New England Journal of Medicine*.

[5] Casalino E, Wolff M, Ravaud P, Choquet C, Bruneel F, Regnier B (2004). Impact of HAART advent on admission patterns and survival in HIV-infected patients admitted to an intensive care unit. *AIDS*.

[6] Narasimhan M, Posner AJ, DePalo VA, Mayo PH, Rosen MJ (2004). Intensive care in patients with HIV infection in the era of highly active antiretroviral therapy. *Chest*.

[7] Vincent B, Timsit J-F, Auburtin M (2004). Characteristics and outcomes of HIV-infected patients in the ICU: impact of the highly active antiretroviral treatment era. *Intensive Care Medicine*.

[8] Wachter RM, Russi MB, Bloch DA, Hopewell PC, Luce JM (1991). Pneumocystis carinii pneumonia and respiratory failure in AIDS: improved outcomes and increased use of intensive care units. *American Review of Respiratory Disease*.

[9] Nichas G, Wachter RM (2000). Outcomes of intensive care for patients with human immunodeficiency virus infection. *Archives of Internal Medicine*.

[10] Afessa B, Green B (2000). Clinical course prognostic factors and outcome prediction for HIV patients in the ICU: the PIP (Pulmonary complications, ICU support, and prognostic factors in hospitalized patients with HIV) study. *Chest*.

[11] Dickson SJ, Batson S, Copas AJ, Edwards SG, Singer M, Miller RF (2007). Survival of HIV-infected patients in the intensive care unit in the era of highly active antiretroviral therapy. *Thorax*.

[12] Powell K, Davis JL, Morris AM, Chi A, Bensley MR, Huang L (2009). Survival for patients with HIV admitted to the ICU continues to improve in the current era of combination antiretroviral therapy. *Chest*.

[13] Chiang H-H, Hung C-C, Lee C-M (2011). Admissions to intensive care unit of HIV-infected patients in the era of highly active antiretroviral therapy: etiology and prognostic factors. *Critical Care*.

[14] Turtle L, Vyakernam R, Menon-Johansson A, Nelson MR, Soni N (2011). Intensive care usage by HIV-positive patients in the HAART era. *Interdisciplinary Perspectives on Infectious Diseases*.

[15] Wachter RM, Luce JM, Hopewell PC (1992). Critical care of patients with AIDS. *Journal of the American Medical Association*.

[16] Marin B, Thiébaut R, Bucher HC (2009). Non-AIDS-defining deaths and immunodeficiency in the era of combination antiretroviral therapy. *AIDS*.

[17] Lau B, Gange SJ, Moore RD (2007). Risk of non-AIDS-related mortality may exceed risk of AIDS-related mortality among individuals enrolling into care with CD_4_
^+^ counts greater than 200 cells/mm^3^. *Journal of Acquired Immune Deficiency Syndromes*.

[18] Shrosbree J, Campbell LJ, Ibrahim F (2013). Late HIV diagnosis is a major risk factor for intensive care unit admission in HIV-positive patients: a single center observational cohort study. *Biomed Central Infectious Diseases*.

[19] Alves C, Nicolás JM, Miró JM (2001). Reappraissal of the aetiology and prognostic factors of severe acute respiratory failure in HIV patients. *European Respiratory Journal*.

[20] Morquin D, Le Moing V, Mura T (2012). Short- and long-term outcomes of HIV-infected patients admitted to the intensive care unit: impact on antiretroviral therapy and immunovirological status. *Annals of Intensive Care*.

[21] Powell K, Huang L (2009). The ART of caring for patients with HIV infection in the ICU. *Intensive Care Medicine*.

[22] Foo H, Clezy K, Post JJ (2012). The long-term outcome of HIV-infected patients after intensive care admission. *International Journal of STD & AIDS*.

[23] Barbier F, Coquet I, Legriel S (2009). Etiologies and outcome of acute respiratory failure in HIV-infected patients. *Intensive Care Medicine*.

[24] Croda J, Croda MG, Neves A, de Sousa dos Santos S (2009). Benefit of antiretroviral therapy on survival of human immunodeficiency virus-infected patients admitted to an intensive care unit. *Critical Care Medicine*.

[25] Japiassú AM, Amâncio RT, Mesquita EC (2010). Sepsis is a major determinant of outcome in critically ill HIV/AIDS patients. *Critical Care*.

[26] Coquet I, Pavie J, Palmer P (2010). Survival trends in critically ill HIV-infected patients in the highly active antiretroviral therapy era. *Critical Care*.

[27] Pathak V, Hurtado Rendon IS, Atrash S (2012). Comparing outcomes of HIV versus non-HIV patients requiring mechanical ventilation. *Clinical Medicine Research*.

[28] Khouli H, Afrasiabi A, Shibli M, Hajal R, Barrett CR, Homel P (2005). Outcome of critically ill human immunodeficiency virus-infected patients in the era of highly active antiretroviral therapy. *Journal of Intensive Care Medicine*.

